# *In vitro* Anti-Inflammatory and Anti-Oxidative Stress Activities of Kushenol C Isolated from the Roots of *Sophora* *flavescens*

**DOI:** 10.3390/molecules25081768

**Published:** 2020-04-12

**Authors:** Byoung Ok Cho, Denis Nchang Che, Ji-Su Kim, Jang Hoon Kim, Jae Young Shin, Hyun Ju Kang, Seon Il Jang

**Affiliations:** 1 Research Institute, Ato Q&A Co., LTD, Jeonju-si, Jeollabuk-do 54840, Korea; sjy8976@naver.com (J.Y.S.); dkgk0608@naver.com (H.J.K.); 2 Department of Health Management, Jeonju University, Jeonju-si, Jeollabuk-do 55069, Korea; chedenis88@gmail.com (D.N.C.); kim1011003@naver.com (J.-S.K.); 3 Department of Food Science and Technology, Chonbuk National University, Jeonju-si, Jeollabuk-do 54896, Korea; 4 Advanced Radiation Technology Institute, Korea Atomic Energy Research Institute, Jeongeup, Jeollabuk-do 56212, Korea; oasis5325@gmail.com

**Keywords:** kushenol C, *Sophora flavescens*, anti-inflammation, anti-oxidative stress

## Abstract

Kushenol C (KC) is a prenylated flavonoid isolated from the roots of *Sophora* *flavescens aiton.* Little is known about its anti-inflammatory and anti-oxidative stress activities. Here, we investigated the anti-inflammatory and anti-oxidative stress effects of KC in lipopolysaccharide (LPS)-stimulated RAW264.7 macrophages, and tert-butyl hydroperoxide (tBHP)-induced oxidative stress in HaCaT cells. The results demonstrated that KC dose-dependently suppressed the production of inflammatory mediators, including NO, PGE_2_, IL-6, IL1β, MCP-1, and IFN-β in LPS-stimulated RAW264.7 macrophages. The study demonstrated that the inhibition of STAT1, STAT6, and NF-κB activations by KC might have been responsible for the inhibition of NO, PGE_2_, IL-6, IL1β, MCP-1, and IFN-β in the LPS-stimulated RAW264.7 macrophages. KC also upregulated the expression of HO-1 and its activities in the LPS-stimulated RAW264.7 macrophages. The upregulation of Nrf2 transcription activities by KC in the LPS-stimulated RAW264.7 macrophages was demonstrated to be responsible for the upregulation of HO-1 expression and its activity in LPS-stimulated RAW264.7 macrophages. In HaCaT cells, KC prevented DNA damage and cell death by upregulating the endogenous antioxidant defense system involving glutathione, superoxide dismutase, and catalase, which prevented reactive oxygen species production from tert-butyl hydroperoxide (tBHP)-induced oxidative stress in HaCaT cells. The upregulated activation of Nrf2 and Akt in the PI3K-Akt signaling pathway by KC was demonstrated to be responsible for the anti-oxidative stress activity of KC in HaCaT cells. Collectively, the study suggests that KC can be further investigated as a potential anti-inflammatory candidate for the treatment of inflammatory diseases.

## 1. Introduction

The roots of *Sophora flavescens aiton* have been used in Chinese traditional medicine as an analgesic, antipyretic, and anthelmintic, and for the treatment of gastrointestinal hemorrhage, diarrhea, and eczema [1]. This prompted the isolation and identification of active compounds of *S. flavescens.* As a result, many prenylated flavonoids with significant biological activities have been identified in *S. flavescens.* Kushenol Z, sophoraflavanone G, and kushenol A were demonstrated to have potent cytotoxicity to lung cancer cells [2]. Kushenol I, kushenol C, kushenol M, leachianone A, and sophoraflavone G were shown to inhibit cytochrome P450 isoform activities in human liver microsomes [3]. Kushenol A and 8-prenylkaempferol exhibited potent tyrosinase inhibitory activities by blocking the conversion of l-tyrosine to l-DOPA by tyrosinase [4]. Despite the well-studied biological activities of *S. flavescens* and its compounds, very little is known about the anti-oxidant and anti-inflammatory activities of the individual active compounds in different cells of the body. However, the anti-inflammatory activities of the crude extracts of *S. flavescens* have been described [5,6,7,8]. 

Inflammation is the normal biological process of the body that occurs when the body is under an external or internal attack. Thus, inflammation is a protective process that protects the body from hazardous stimuli-like infections, injuries, and oxidative stress [9]. Normally, after the infection or injury has been resolved, it is expected that the inflammatory process will stop, as the body has been healed of the infection or injury. However, this is not the situation in some cases in which the inflammatory process continues even after the healing process is completed, thereby resulting in excessive or even chronic inflammation [10]. This excessive or chronic inflammation will further cause painful diseases, such as asthma, inflammatory bowel diseases, atopic dermatitis, rheumatoid arthritis, colitis, systemic lupus erythematosus, and autoimmune diseases [11]. The inflammation will be caused by the recruitment of various inflammatory cells, including macrophages and lymphocytes that will secrete a vast array of inflammatory mediators, such as nitric oxide, interleukin (IL)-1β, IL-4, IL-5, IL-6, tumor necrosis factor-alpha (TNF-α), prostaglandin E2 (PGE_2_), and interferon-gamma (IFNγ) [12,13]. Additionally, oxidative stress generates reactive oxygen species (ROS) that activate the MAPK-signaling pathway and induce AP-1 and NF-κB-mediated expression and production of inflammatory cytokines, which boosts inflammation [14,15]. Therefore, it is important to regulate the inflammatory process to prevent the development of inflammatory diseases. Several drugs have been used to treat excessive or chronic inflammation, but these come with some adverse side effects that exceed their benefits in some patients [16]. For example, glucocorticoids widely used as anti-inflammatory drugs have several adverse side effects, including fluid retention, high blood pressure, headache, muscle weakness, facial hair growth, puffiness of the face (moon face), thinning skin/easy bruising, and slow wound healing [17]. This has led to the intensification of research for the development of alternative anti-inflammatory agents with little or no side effects possible from natural origins. In the present study, we investigated the anti-inflammatory and anti-oxidative stress effects of kushenol C in a macrophage and skin cell lines and clarify the mechanism of action.

## 2. Material and Methods

### 2.1. Materials

Kushenol C (KC) was a gift from Dr. Jang Hoon Kim of the Korea Atomic Energy Research Institute (Jeongeup, Korea). Dulbecco’s modified Eagle medium (DMEM) and fetal bovine serum were purchased from Gibco, Grand Island, NY, USA. Penicillin/streptomycin antibiotics came from Invitrogen, Carlsbad, CA, USA. EZ-Cytox reagent and EZ-western Lumi Pico Alpha were obtained from DoGenBio, Seoul, Korea. Greiss reagent, protease inhibitors, phosphatase inhibitors, tert-butyl hydroperoxide (tBHP), and lipopolysaccharide (LPS) were purchased from Sigma-Aldrich (St. Louis, MO, USA). Radio-immunoprecipitation assay buffer (RIPA buffer) and the NE-PER nuclear and cytoplasmic extraction reagent came from Thermo Scientific, Rockford, IL, USA. Bio-Rad Protein Assay came from Bio-Rad, Hercules, CA, USA. STAT 1 and 6, p-STAT 1 and 6, p-Akt, Akt (Cell Signaling Technology Inc, Beverly, MA, USA); p-NF-κB, HO-1, Nrf2, Sirt, OGG1, HRP conjugated secondary antibody (Santa Cruz Biotechnology, Santa Cruz, CA., USA), and actin (Biosciences, Franklin Lakes, NJ, USA) antibodies were used in this study. Enhanced chemiluminescence detection system kit came from Amersham Biosciences, Piscataway, NJ, USA. GSH, SOD, and catalase came from Cayman Chemical, Ann Arbor, MI, USA. HO-1 activity kit was purchased from Cusabio (Baltimore, MD, USA). TransAM® Nrf2 and NF-κB Transcription Factor ELISA kit were purchased from Active Motif (Carlsbad, CA, USA). IFN-β, PGE2, IL-6, IL-1β, and MCP-1 ELISA kit came from R&D Systems (Minneapolis, MN, USA). SnPP and carboxy-H2DCFDA probe came from Porphyrin Products Inc. (Logan, UT, USA) and Invitrogen (Carlsbad, CA, USA), respectively.

### 2.2. Cell Culture

RAW 264.7 and HaCaT cell lines were purchased from ATCC, Manassas, VA, USA. Both cells were cultured and maintained in DMEM solution supplemented with 10% fetal bovine serum, 100 U/mL penicillin, and 100 μg/mL streptomycin in a 5% CO_2_ incubator at 37 °C. 

### 2.3. Cell Viability

The cell viability assay was carried out using EZ-Cytox reagent. The RAW 264.7 cells (2 × 10^5^ cells/mL) and HaCaT cells (1 × 10^5^ cells/mL) were seeded in 96-well plates and incubated for 24 h. The RAW 264.7 cells were then treated with KC (0, 12.5, 25, 50, or 100 µM), while the HaCaT cells were treated with KC (0, 10, 20, 30, 40, or 50 µM) for another 24 h, after which 0.01 mL of EZ-Cytox reagent was added to each well. The absorbance in each well was subsequently measured after 4 h at 450 nm with a microplate reader (Tecan, Männedorf, Switzerland). The absorbance corresponded with the number of viable cells.

### 2.4. Cell Proliferation Assay

HaCaT cell proliferation was measured using the EZ-Cytox reagent. The cells (1 × 10^5^ cells/mL) were seeded in a 96-well plate for 24 h. The cells were then pretreated with 10, 30, and 50 µM of KC for 1 h and subsequently stimulated with 1 mM of tBHP for 6 h. After the 6 h, 0.01 mL of EZ-Cytox reagent was added to each well and they further incubated for 4 h. The absorbance in each well was then measured at 450 nm with a microplate reader (Tecan). The absorbance corresponded with the number of viable cells.

### 2.5. Nitric Oxide Production Assay

**Figure 1:**
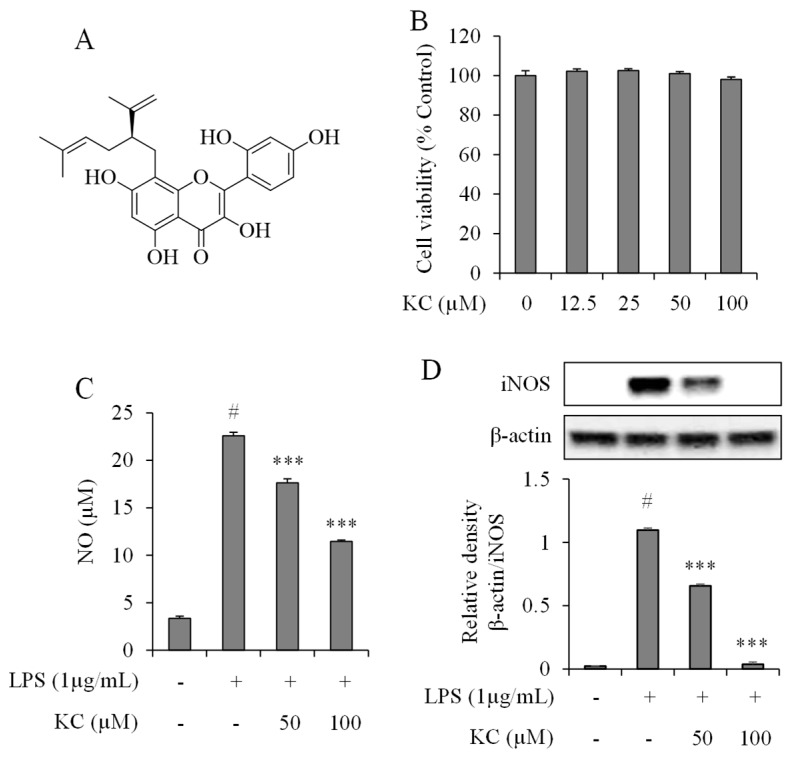
The chemical structure of kushenol C (**A**), and the effects of kushenol C (KC) on cell viability (**B**), NO production (**C**), and iNOS expression levels (**D**) in LPS-stimulated RAW264.7 macrophages. (**B**) RAW264.7 cells were treated with KC (0, 12.5, 25, 50, or 100 μM) for 24 h, and the relative cell viability was assessed by WST-1 assay. (**C**) RAW264.7 cells were pretreated with KC (50 or 100 μM) and stimulated with LPS (1 µg/mL). The culture supernatant was subjected to a nitrite assay. (**D**) The iNOS expression levels were determined by western blot analysis of total protein extract. Error bars represent the means ± SDs, #*p* < 0.001 vs. control, ***p < 0.001 vs. LPS-only treated cells.

RAW264.7 cells (2 × 10^5^ cells/mL) were cultured in a 6-well cell culture dish for 24 h and pretreated with KC at concentrations of 50 and 100 µM for 1 h. Then the cells were stimulated by treating the cells further with 1 µg/mL of LPS for 16 h (Figure 1C). For the potent NO inhibitor assay, the cells were pretreated with or without 100 µM of KC or 30 µM of SnPP for 1 h before stimulation with the LPS. The NO secretion was measured by Griess method. In brief, 100 μL of a cell media supernatant was added to 100 µL of Greiss reagent and incubated for 15 min at room temperature. The absorbance was then determined at 540 nm. The concentration of NO was determined from a sodium nitrite standard curve developed during the experiment. 

### 2.6. ELISA

The RAW264.7 cells (2 × 10^5^ cells/mL) were cultured in a 6-well cell culture dish for 24 h and pretreated with KC at concentrations of 50 and 100 µM for 1 h. Then the cells were stimulated by treating the cells with 1 µg/mL of LPS for 16 h. The production of PGE_2_, IL-6, IL-1β, MCP-1, and IFN-β in the cell culture supernatants was measured using ELISA kits according to the manufacturer’s instructions. Samples were assayed in triplicate.

### 2.7. Total Protein Extraction

In RAW 264.7 cells (2 × 10^5^ cells/mL), total protein was extracted from the cells pretreated with 50 and 100 µM of KC for 1 h and stimulated with 1 µg/mL of LPS for 4 h or 20 h (Figure 1D). In HaCaT cells (2 × 10^5^ cells/mL), total protein was extracted from the cells pretreated with 50 µM of KC for 1 h and stimulated with or without 1 mM of tBHP for 6 h. For total protein extraction, the cells were lysed with RIPA in the presence of protease and phosphatase inhibitors and centrifuged at 12,000 rpm for 15 min at 4 °C to obtain the supernatant containing the whole-cell protein extract. The extracts were stored at −80 °C until they were used for further studies. The protein contents were determined by a Bio-Rad Protein Assay.

### 2.8. Cytosol and Nuclear Protein Extraction

RAW264.7 cells (2 × 10^5^ cells/mL) were cultured in a 100 mm cell culture dish for 24 h and pretreated with 50 and 100 µM of KC for 1 h and stimulated with 1 µg/mL of LPS for 0.5 h. Nuclear and cytosol proteins were extracted using the NE-PER nuclear and cytoplasmic extract kit following the manufacturer’s instructions. For cytoplasmic protein extraction, the cells were harvested and washed in ice-cold PBS. After centrifugation of the cells at 500× *g* for 2 min at 4 °C, the cells were resuspended in 0.1 mL of ice-cold cytoplasmic extraction reagent I. The tubes were vigorously vortexed and incubated on ice for 10 min. Then, 0.0055 mL of cytoplasmic extraction reagent II was added and further incubated for 1 min on ice. This was followed by vortexing and centrifugation at 16,000× *g* for 5 min at 4 °C to obtain the supernatant as the cytoplasmic fraction. The cytoplasmic fractions were stored at −80 °C. For nuclear protein extraction, the pellets obtained following the cytoplasmic extraction were resuspended in 0.05 mL of nuclear extraction reagent and vigorously vortexed at 10 min intervals for a total of 40 min on ice. This was followed by centrifugation at 16,000× *g* for 15 min at 4 °C to obtain the supernatant as the nuclear fraction. The nuclear fractions were stored at −80 °C until use. The protein concentrations in the cytoplasmic and nuclear extract were evaluated by using Bio-Rad Protein Assay. The purity of the nuclear fraction was determined by the absence of actin band following a western blot experiment. 

### 2.9. Western Blotting 

Thirty micrograms of whole-cell proteins from each sample were separated on sodium dodecyl sulfate (SDS) polyacrylamide gels and transferred to polyvinylidene difluoride (PVDF) membranes. The membranes were blocked with 5% bovine serum albumin (BSA) in Tris-buffered saline, containing 0.1% Tween (TBS-T) buffer for 1 h at room temperature and incubated overnight with primary antibody dilutions a 4 °C with gentle shaking. Following incubation with the primary antibodies, the membranes were washed in 5 changes of TBST (5 min for each wash) and incubated with HRP conjugated secondary antibody for 2 h at room temperature. Following incubation with the secondary antibodies, the membranes were washed in 5 changes of TBST (5 min for each wash) and the protein bands were detected using an enhanced chemiluminescence detection system (Amersham Biosciences, Piscataway, NJ, USA). To ensure equal protein loading, the membranes were stripped and reprobed with anti β-actin antibodies using the protocol described above. The density of each band in an immunoblot was analyzed using the ImageJ gel analysis software (developed by the National Institutes of Health, Bethesda, MD, USA).

### 2.10. GSH, SOD, HO-1, and Catalase Activity Assay 

The GSH, SOD, and catalase activities in HaCaT cells and HO-1 activity in RAW267.4 cells were measured from the whole-cell proteins. The experiments were performed per the manufacturer’s procedure. 

### 2.11. NF-κB and Nrf2 Activity Assay

The NF-κB and Nrf2 DNA-binding activities were measured in the nuclear protein extracts using the NF-κB transcription factor and Nrf2 transcription factor assay kits. Nuclear extracts (10 µg) and complete binding buffer (0.03 mL) were added into wells pre-fixed with NF-κB p65 or Nrf2 DNA target and incubated for 1 h at room temperature with mild agitation. Following the incubation, the wells were washed three times with wash buffers, and 0.1 mL of diluted NF-κB or Nrf2 antibodies, which recognize only p65 or Nrf2 epitopes that are bound to DNA in the wells, was added to each well and incubated for 1 h at room temperature. Then, the wells were washed as above, and HRP-conjugated antibodies (0.1 mL) were added to each well and incubated for 1 h at room temperature. Following incubation with the HRP-conjugated antibodies, the wells were washed as described above; 0.1 mL of developing solution was added into the wells for 5 min; and then, the reaction was stopped by adding 0.1 mL of stopping solution. The absorbance, which corresponded with the DNA binding activities, was read at 450 nm using a spectrophotometer (Tecan, Männedorf, Switzerland)

### 2.12. Intracellular ROS Measurement

Intracellular ROS concentration was measured using a carboxy-H_2_DCFDA probe. HaCaT cells (1 × 10^5^ cells/mL) were cultured in 6-well cell culture plates for 24 h and pretreated with 50 µM of KC for 1 h and stimulated with 1 mM of tBHP for 1 h. The cells were further incubated with 10 µM carboxy-H_2_DCFDA for 20 min. Following the incubation, the cells were washed, harvested, and immediately examined using a flow cytometer (Cytomics FC500; Beckman, Miami, FL, USA).

### 2.13. Statistical Analysis

All data were analyzed using the SPSS statistics program (version 22 SPSS Inc., Chicago, IL, USA). All data are presented as means ± SDs. Differences between the variables were reported using a one-way analysis of variance. Duncan’s multiple range tests (*p*-value < 0.05) were used to compare the mean values.

## 3. Results

### 3.1. Evaluation of NO Production and iNOS Expression Levels in LPS-Stimulated RAW264.7 Cells Treated with KC

To determine the effects of KC on NO production and iNOS expression in the LPS-stimulated RAW264.7 cells, we first carried out cell viability studies to determine the cytotoxic effects of KC administration in RAW264.7 cells. We treated the cells with different concentrations of KC (0, 12.5, 25, 50, and 100 μM) for 24 h, and the cell cytotoxicity was determined. The results demonstrated that KC did not cause cytotoxicity to the RAW264.7 cells up to 100 μM (Figure 1B). From the above results, we chose concentrations of 50 and 100 μM of KC to study their anti-inflammatory effects in stimulated RAW cells. Then, we investigated the effects of KC administration on NO production in the stimulated cells. We found that cells treated with only LPS had significantly raised NO production, while cells pretreated with 50 or 100 μM of KC before administration of LPS showed a significant and dose-dependent decrease in NO production when compared to the LPS only-treated cells (Figure 1C). We also examined iNOS expression in the cytosol of the cells. The results showed that LPS-only treated cells had increased expression of iNOS, while the cells pretreated with 50 or 100 μM of KC before administration of LPS showed a significant and dose-dependent decrease in iNOS expression when compared to the LPS only-treated cells (Figure 1D). 

### 3.2. Evaluation of PGE_2_, IL-6, IL-1β, IFN-β, and MCP-1 Production in LPS-Stimulated RAW264.7 Cells Treated with KC

**Figure 2:**
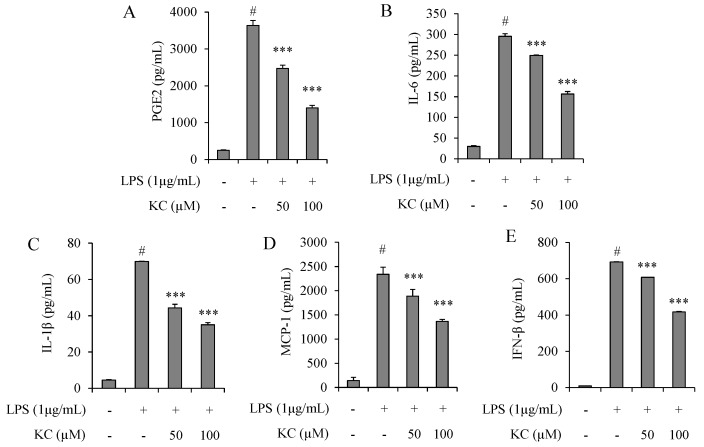
The effects of KC on PGE_2_ (**A**), IL-6 (**B**), IL-1β (**C**), MCP-1 (**D**), and IFN-β (**E**) production in LPS-stimulated RAW264.7 macrophages. RAW264.7 cells were pretreated with KC (50 or 100 μM) and stimulated with LPS (1 µg/mL). The culture media supernatants were collected and subjected to ELISA. Error bars represent the means ± SDs, #*p* < 0.001 vs. control, ***p < 0.001 vs. LPS-only treated cells.

Next, we determined the effects of KC on the production of pro-inflammatory mediators, suh as PGE_2_, IL-6, IL-1β, IFN-β, and MCP-1 in stimulated RAW264.7 cells. The results demonstrated that cells treated with 1 µg/mL of LPS only significantly raised the levels of PGE_2_, IL-6, IL-1β, IFN-β, and MCP-1 concentrations in the cell culture media, when compared to the untreated cells (Figure 2). However, the concentrations of PGE_2_, IL-6, IL-1β, IFN-β, and MCP-1 were decreased in a dose-dependent manner in cells pretreated with 50 or 100 μM of KC before stimulation with LPS (Figure 2).

### 3.3. Evaluation of STAT and NF-κB Pathways in LPS-Stimulated RAW264.7 Cells Treated with KC

**Figure 3:**
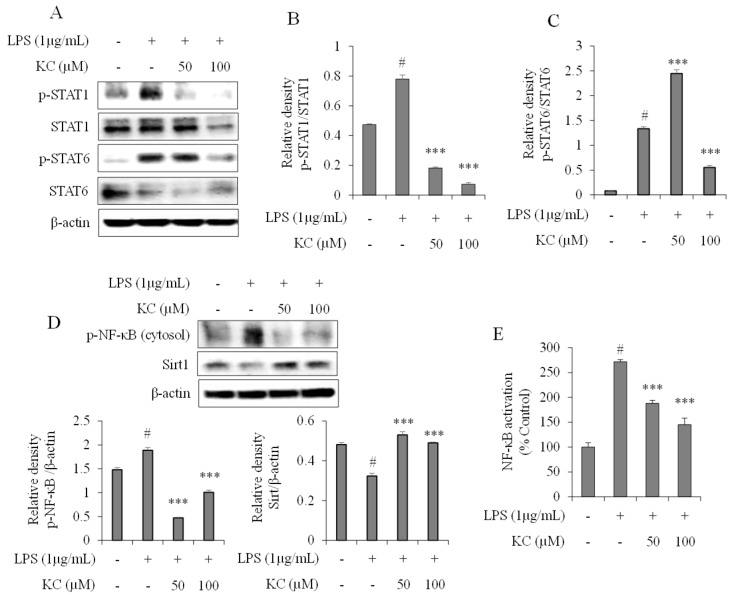
The effects of KC on LPS-induced STAT1 and STAT6 activation (**A**–**C**), NF-κB activation (**D**), and NF-κB-DNA binding activities in RAW264.7 cells. RAW264.7 cells were pretreated with KC (50 or 100 μM) and stimulated with LPS (1 µg/mL). The expressions of STAT1, STAT6, p-STAT1, p-STAT6, p-p65, and Sirt1 (**A**–**D**) were determined by western blot analysis, and the bands developed were analyzed using ImageJ software. DNA binding activity of NF-κB was measured using an ELISA-based method (**E**). Error bars represent the means ± SDs, #*p* < 0.001 vs. control, ***p < 0.001 vs. LPS-only treated cells.

Next, we determined the effects of KC on STAT1 and STAT6 activation in stimulated RAW264.7 cells. As shown in Figure 3A–C, when the cells were treated with 1 µg/mL of LPS alone, STAT1 and STAT6 phosphorylation were significantly increased 4 h after treatment. However, when the cells were pretreated with 50 or 100 μM of KC before stimulation with LPS, the phosphorylation of both STAT1 and STAT6 was significantly decreased in a dose-dependent manner. 

We also investigated whether KC affected the expression of SIRT1, phosphorylation of NF-κB and its transcriptional activities in the stimulated RAW264.7 cells. The results revealed that cells treated with LPS alone significantly lowered the expression of SIRT1 and concomitantly increased the expression of phosphorylated NF-κB. However, the expression of SIRT1 and phosphorylated NF-κB were reversed when the cells were pretreated with 50 or 100 μM of KC before stimulation with the LPS (Figure 3D). The results of the NF-κB transcription activities demonstrated that cells treated with LPS alone significantly increased NF-κB-DNA binding activities, while the NF-κB-DNA binding activities in cells pretreated with 50 or 100 μM of KC before stimulation with the LPS were significantly decreased in a dose-dependent manner (Figure 3E).

### 3.4. Evaluation of HO-1 Induction and Nrf2 Activation in LPS-Stimulated RAW264.7 Cells Treated with KC

**Figure 4:**
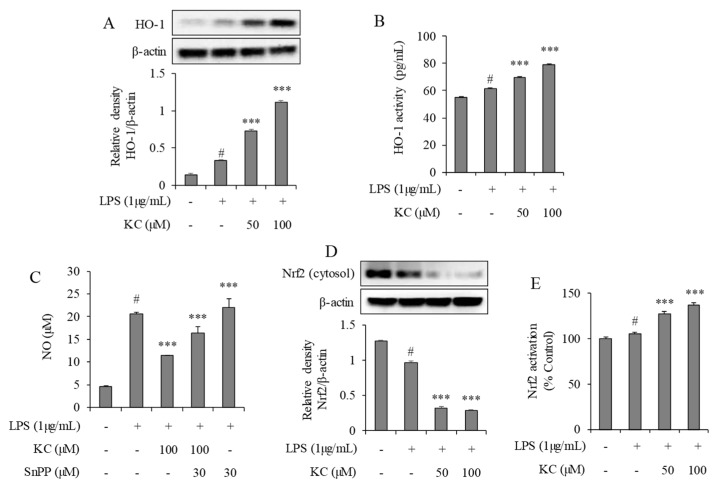
The effects of KC on HO-1 expression in LPS-stimulated RAW264.7 cells. RAW264.7 cells were pretreated with KC (50 or 100 μM) and stimulated with LPS (1 µg/mL). The expression of HO-1 (**A**) was investigated by western blot analysis using the total protein extract, and HO-1 activity (**B**) was investigated using an ELISA-based method in nuclear protein extracts. The effects of HO-1 inhibition were investigated by measuring NO production in the presence of HO-1 inhibitor (SnPP) in the stimulated cells (**C**). The expression of Nrf2 (**D**) was investigated by western blot analysis in total protein extracts, and the DNA binding activity of Nrf2 (**E**) was measured using an ELISA-based method nuclear protein extracts. Error bars represent the means ± SDs, # *p* < 0.001 vs. control, *** *p* < 0.001 vs. LPS-only treated cells

The effect of KC on HO-1 expression was investigated in the RAW264.7 stimulated cells. We demonstrated that cells treated with 1 µg/mL of LPS alone significantly increased HO-1’s expression and activity (Figure 4) when compared to the non-treated cells. However, HO-1’s expression (Figure 4A) and activity (Figure 4B) were further increased when the cells were pretreated with 50 or 100 μM of KC before stimulation with the LPS. We also investigated the possibilities of whether HO-1 induction was responsible for the KC-mediated inhibition of NO production in the stimulated cells. We treated the cells with LPS and KC in the presence of SnPP, a selective inhibitor of HO-1. We found that the cells treated with LPS alone significantly increased the NO production, as expected, and that the cells pretreated with KC at 100 μM before stimulation with LPS decreased NO production. Interestingly, when the cells were pretreated with KC at 100 μM and SnPP at 30 μM, NO production was restored, while cells treated with SnPP alone before LPS treatment did not significantly affect the NO production (Figure 4C).

We also investigated the expression pattern and transcription activities of Nrf2 in the LPS-stimulated cells. In the cytosols of the cells, we found that cells treated with 1 µg/mL of LPS alone significantly decreased Nrf2 expression when compared to the untreated cells. In addition, cells that were pretreated with 50 or 100 μM of KC before stimulation with the LPS significantly decreased the Nrf2 expression when compared to the non-treated cells or LPS-only treated cells (Figure 4D). Nrf2-DNA binding activity results also demonstrated that cells pretreated with 50 or 100 μM of KC before stimulation with the LPS significantly increased the Nrf2–DNA activity when compared to the LPS only-treated cells (Figure 4E).

### 3.5. Evaluation of KC Treatment on Oxidative Stress in HaCaT Cells

**Figure 5:**
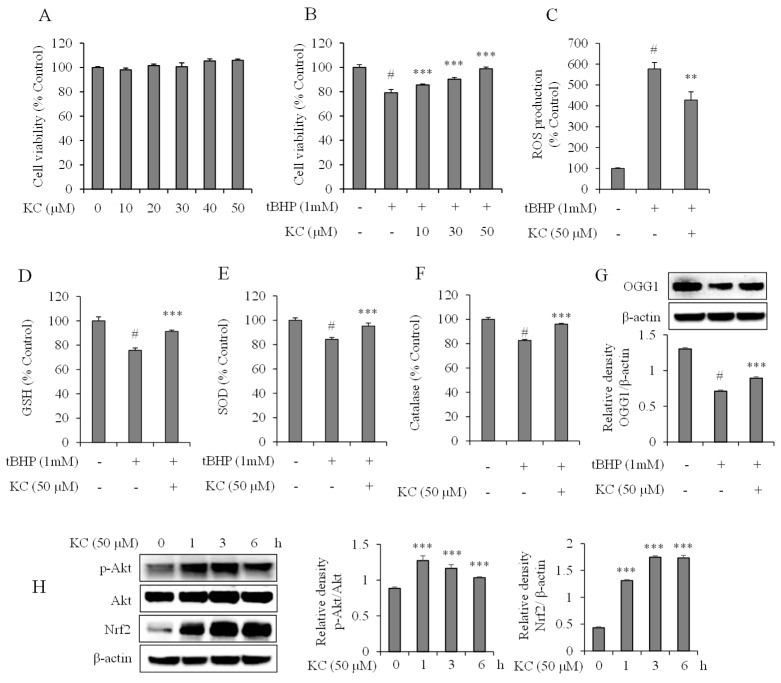
The effect of KC on tBHP-induced oxidative stress in HaCaT cells. HaCaT cells were treated with KC (0, 10, 20, 30, 40, or 50 μM) for 24 h, and the relative cell viability was assessed by WST-1 assay (**A**). HaCaT cells were pretreated with KC (10, 30, or 50 μM) and stimulated with 1 mM of tBHP and the cell death was investigated (**B**). ROS production (**C**) in HaCaT cells were investigated using a carboxy-H_2_DCFDA. GSH (**D**); SOD (**E**); catalase (**F**); OGG1 (**G**); and p-Akt, Akt, and Nrf2 (**H**) were investigated in whole-cell extracts. Error bars represent the means ± SDs, # *p* < 0.001 vs. control, ** *p* < 0.01, *** *p* < 0.001 vs. tBHP-only treated cells (**B**–**G** or vs. 0 h cells **H**).

The effects of KC on oxidative stress in HaCaT cells were also studied. To start with, we investigated the cytotoxic effects of KC on HaCaT cells. We treated the cells with different concentrations of KC (0, 10, 20, 30, 40, and 50 μM) for 24 h and determined the cell viability. The results demonstrated that KC did not cause significant cytotoxicity to the HaCaT cells up to 50 μM (Figure 5A). Then we investigated the effects of KC on tBHP-induced oxidative stress, which leads to cell death. We found that the cell viability was significantly decreased in cells that were treated with 1 mM of tBHP alone, and that the cell viability was dose-dependently and markedly increased in cells that were pretreated with 10, 30, or 50 μM of KC before treatment with the tBHP when compared to the tBHP-only treated cells (Figure 5B).

The scavenging ability of KC against intracellular ROS generation in the cells was explored. The results revealed that intracellular ROS levels were significantly increased in cells treated with the tBHP only. However, the intracellular ROS levels were significantly decreased in the cells pretreated with 50 μM of KC before treatment with the tBHP (Figure 5C). 

The activation of GSH, SOD, and catalase by KC in HaCaT cells was also investigated. The results revealed that G SH, SOD, and catalase levels were significantly decreased when the cells were treated with tBHP alone. However, the cells that were pretreated with 50 μM of KC before treatment with the tBHP showed a significant increase in GSH, SOD, and catalase levels, compared to the tBHP-only treated cells (Figure 5D–F). Further findings revealed that cells treated with tBHP alone significantly decreased the expression of OGG1, when compared to the untreated cells, while OGG1 expressions were significantly increased in cells that were pretreated with 50 μM of KC before treatment with tBHP, when compared to the tBHP-only treated cells (Figure 5G).

### 3.6. Evaluation of KC Treatment on Nrf2 and Akt Expression in HaCaT Cells

We measured the protein expression levels of Nrf2 and Akt in HaCaT cells following treatment with KC at 50 μM at different time intervals (0, 1, 2, 3, and 6 h). We found that treatment of the cells with KC increased the expression levels of Nrf2 and phosphorylated Akt, which had no effects on Akt (Figure 5H).

## 4. Discussion

In the present study, we investigated the anti-inflammatory and anti-oxidative stress effects of KC isolated from roots of *S. flavescens* in LPS-stimulated RAW264.7 and tBHP-stimulated HaCaT cells, respectively. LPS is a well-known stimulator of macrophages which causes macrophages to produce and secrete pro-inflammatory mediators, such as NO, PGE_2_, TNF-α, IL-6, and IL-1β [18]. They act through their TLR4 receptors on the surfaces of macrophages to activate signaling pathways, such as NF-κB, MAPK, and STAT pathways, which are responsible for the activation of inflammatory genes [19,20,21]. NO production is mediated by iNOS expression, and its production has been linked with many inflammatory diseases that damage normal tissues [22]. Thus, suppressing its expression is deemed necessary for the treatment of inflammatory diseases. Previous studies have reported that several phytochemicals attenuate iNOS expression and NO production [23,24]. Consistent with previous reports, our results demonstrated that LPS-stimulated RAW264.7 cells increase iNOS expression, and hence, NO production and secretion. The production of NO in the stimulated cells was inhibited by KC, probably through its suppression of iNOS expression. 

PGE_2_, IL-6, IFN-β, and IL-1β have been implicated in inflammatory and autoimmune diseases, including rheumatoid arthritis and systemic lupus erythematous [25,26,27]. IL-1β is specifically associated with acute and chronic inflammation, and has emerged as a therapeutic target for an expanding number of systemic and local inflammatory conditions called autoinflammatory diseases [28]. IFN-β, is also known to sensitize cells to TLR agonists, thereby boosting the releases of inflammatory mediators [29]. MCP-1 by regulating the migration and infiltration of monocytes/macrophages also plays an important role in inflammatory diseases [30]. Thus, regulating these inflammatory mediators is important in the prevention and treatment of inflammatory diseases. Research had shown that extracts and compounds of *S. flavescens* suppress pro-inflammatory cytokines, including TNF-α, IL-6, and IL-1β, and cyclooxygenase-2 in many cell lines [7,31,32]. In the present study, we demonstrated that PGE_2_, IL-6, IFN-β, MCP-1, and IL-1β secretions were decreased in the stimulated RAW264.7 cells treated with KC. These results meant that KC suppressed the production and secretion of these inflammatory mediators, which is consistent with the previous studies. Besides, as KC suppressed the secretion of the chemoattractant molecule-MCP-1, we suggested that the KC could also regulate inflammation by modulating the recruitment of inflammatory cells to the inflammation site in vivo. Therefore, our findings suggest that KC has an anti-inflammatory effect that might have inhibited iNOS-mediated NO production, and PGE_2_, IL-6, IFN-β, MCP-1, and IL-1β production.

To understand the mechanism of the action of KC in inhibiting the production of NO, PGE_2_, IL-6, IFN-β, and MCP-1, and the IL-1β production in stimulated RAW264.7 cells, we examined the activation of STAT1, STAT6, and NF-κB following KC treatment of stimulated cells. As earlier explained, NF-κB plays an important role in many aspects of human diseases and is an important transcription factor involved in inflammatory gene expression and cytokine production. Here, we investigated whether KC modulated the activation of NF-κB and influenced NF-κB-DNA binding activities. The fact that the activated form of NF-κB (phosphorylated NF-κB) and the DNA binding ability of NF-κB were found to be decreased in the KC treated RAW 264.7 cells stimulated by LPS meant that KC had inhibited the activation and DNA binding activities of NF-κB, which could be partly responsible for the inhibition of the inflammatory mediators released by the stimulated RAW 264.7 cells. Additionally, the fact that SIRT1 was also found to be activated in KC-treated cells might have contributed to the inhibition of NF-κB-regulated inflammatory gene expression, as SIRT1 is known to deacetylate the RelA/p65 subunit of NF-κB, thereby preventing its activation [33]. In addition, KC also inhibited the activation of STAT1 and STAT6, as phosphorylated forms of STAT1 and STAT6 were found to be decreased in the KC-treated RAW264.7 cells stimulated by LPS. STAT1 and STAT6 have been reported to be increasingly expressed in rheumatoid arthritis, wherein they mediate the secretion of inflammatory mediators [34,35]. It is also possible that the action of KC on STAT1 activation might be linked to the suppression of LPS-induced IFN-β, as IFN-β is a principle STAT1-activating cytokine [36]. These findings suggested that the anti-inflammatory activities of KC might have been mediated by the inhibition of NF-κB and STAT activation by KC.

HO-1 is widely expressed in inflammatory diseases. HO-1 breaks the heme in hemoglobin into CO, iron, and biliverdin. Biliverdin is further converted to bilirubin, which is an endogenous antioxidant [37]. In activated macrophages, it was reported that the by-products of heme inhibited the production of pro-inflammatory cytokines such as TNF-α, IL-1β, and IL-6 [38]. The study suggested that increase in HO-1 expression during inflammation was beneficial for the treatment of inflammatory diseases. Here, we found that KC further enhanced HO-1 expression and activity in LPS-stimulated RAW264.7 cells. To prove that HO-1 exerts anti-inflammatory activities, and the beneficial effects of KC, we pretreated the RAW264.7 cells with KC and/or SnPP, an HO-1 inhibitor, and found that treatment with KC alone prevented NO production, while treatment with SnPP alone had no effects on NO production. Moreover, treatment with both KC and SnPP inhibited NO production but not compared to KC treatment alone. This meant that KC inhibited NO production partly by upregulating the expression of HO-1 in RAW264.7 cells. The Nrf2 is a transcription factor that regulates expression of HO-1 [39]. Further findings demonstrated that Nrf2 was decreased in the cytosols of KC-treated LPS-stimulated RAW 264.7cells, but their DNA-binding activities were concomitantly increased, implying that KC’s mediated increase in HO-1 expression was as a result of its action on Nrf2. Taken together, these results suggested that KC’s action on Nrf2-induced HO-1 expression partially contributed to its anti-inflammatory activity. 

Oxidative stress is another cause of inflammation in many inflammatory diseases. It is caused by an increase in the production of ROS to an extent that the endogenous antioxidant defense system cannot handle, resulting in chronic inflammation [40]. The main targets of the products of oxidative stress are proteins, lipids, and DNA/RNA, thereby causing irreversible damage and cell death [41,42]. Thus, the prevention of oxidative stress should be regarded as one of the key targets for the treatment of inflammatory diseases. tBHP has been widely used in in vitro models to investigate oxidative stress [43,44]. Here, we demonstrated that tBHP caused oxidative stress and DNA damage in HaCaT cells, as cell viability was decreased followed by an increase in the production of ROS; down-regulation of the endogenous antioxidant defense system; and a decrease in OGG1-a marker of DNA damage. KC, at non-cytotoxic doses (95% CI), prevented cell death and DNA damage probably by up-regulating the endogenous antioxidant defense system and preventing ROS production, as GSH, SOD, and catalase were found to be increased in the KC-treated cells. To determine whether the anti-oxidative stress effects of KC were due to the regulation of the endogenous antioxidant defense system, we investigated the effects of KC on Nrf2 and Akt activation in HaCaT cells in normal conditions. We found that under normal conditions without oxidative stress, KC could increase the expression of Nrf2 and activate Akt. Antioxidant enzymes are induced by Nrf2 activation via the PI3K–Akt signaling pathway [45]. The fact that KC activated Akt and increased the expression of Nrf2, meant that the action up-regulated the endogenous antioxidant defense system, resulting in a decrease in ROS production, which causes DNA damage and cell death. The results are consisten5 with other studies which reported that polyphenolic compounds protect cells inducing antioxidant enzyme expression via PI3K mediated Nrf2 activation [46,47,48]. Taken together the results, we can conclude that KC also prevented oxidative stress in HaCaT cells, thereby contributing to its anti-inflammatory effect.

In conclusion, KC, isolated from the roots of *S. flavescens* suppresses the production of NO; iNOS; and other pro-inflammatory mediators, including PGE_2_ IL-6, IL1-β, MCP-1, and IFN-β, in LPS-stimulated RAW264.7 macrophages. KC also protects against tBHP-induced oxidative stress and cell death via up-regulating the endogenous antioxidant defense system and scavenging ROS in HaCaT cells. The study suggests that KC should be further investigated as a potential anti-inflammatory candidate for the prevention and treatment of inflammatory diseases.
